# Aurora Kinase A Is Not Involved in CPEB1 Phosphorylation and *cyclin B1* mRNA Polyadenylation during Meiotic Maturation of Porcine Oocytes

**DOI:** 10.1371/journal.pone.0101222

**Published:** 2014-07-01

**Authors:** Pavla Komrskova, Andrej Susor, Radek Malik, Barbora Prochazkova, Lucie Liskova, Jaroslava Supolikova, Stepan Hladky, Michal Kubelka

**Affiliations:** 1 Institute of Animal Physiology and Genetics, Academy of Sciences of the Czech Republic, Libechov, Czech Republic; 2 Institute of Molecular Genetics, Academy of Sciences of the Czech Republic, Prague, Czech Republic; Institut de Génétique et Développement de Rennes, France

## Abstract

Regulation of mRNA translation by cytoplasmic polyadenylation is known to be important for oocyte maturation and further development. This process is generally controlled by phosphorylation of cytoplasmic polyadenylation element binding protein 1 (CPEB1). The aim of this study is to determine the role of Aurora kinase A in CPEB1 phosphorylation and the consequent CPEB1-dependent polyadenylation of maternal mRNAs during mammalian oocyte meiosis. For this purpose, we specifically inhibited Aurora kinase A with MLN8237 during meiotic maturation of porcine oocytes. Using poly(A)-test PCR method, we monitored the effect of Aurora kinase A inhibition on poly(A)-tail extension of long and short cyclin B1 encoding mRNAs as markers of CPEB1-dependent cytoplasmic polyadenylation. Our results show that inhibition of Aurora kinase A activity impairs neither *cyclin B1* mRNA polyadenylation nor its translation and that Aurora kinase A is unlikely to be involved in CPEB1 activating phosphorylation.

## Introduction

Cytoplasmic polyadenylation is a well-documented regulatory mechanism, which governs the translation of maternal mRNAs in the oocytes of different species. This enables the time- and space-specific translation of the most important signaling molecules for the meiotic cell cycle, e.g. cyclins and c-mos [1]. Messenger RNAs of such molecules contain in their 3′-untranslated region (3′UTR) at least two *cis*-factors: the cytoplasmic polyadenylation element (CPE) and the polyadenylation signal, hexanucleotide AAUAAA [2].

The CPE binds the CPE-binding protein 1 (CPEB1), which functions as a translational activator or repressor according to its phosphorylation state. The non-phosphorylated CPEB1 represses the translation initiation of CPE-containing mRNAs by recruiting other *trans*-acting factors such as Maskin and Pumilio [3]–[5]. On the contrary, the CPEB1 phosphorylation at Ser174 (in *Xenopus* oocytes, analogous to Thr172 in porcine oocytes) activates the polyadenylation of a CPE-containing mRNA by excluding the poly(A)-specific ribonuclease (PARN) from its 3′UTR [6]. The second wave of CPEB phosphorylation depends on polo-like kinase 1 (PLK1) and cyclin-dependent kinase 1 (CDK1), also aided by PIN1, and leads to the degradation of CPEB1 by the ubiquitin-proteasome pathway [7]-[11]. The partial degradation of CPEB1 is thought to be necessary for the cytoplasmic polyadenylation of mRNAs, which contain two or more CPEs in their 3′UTR [7], [12].

Aurora kinase A (AURKA) has been previously proposed to be the kinase responsible for the polyadenylation-activating CPEB1 phosphorylation in *Xenopus* [13] and mouse oocytes [14]. Beside this possible function, other key roles of AURKA have been described both during mitotic and meiotic cell cycles, suggesting that it is responsible for the centrosome maturation and separation, proper spindle formation, and chromosome segregation [15]-[19]. However, the role of AURKA in CPEB1 activation has been recently questioned. Keady et al. [20] showed that in *Xenopus* oocyte extracts the phosphorylation of CPEB1 at Ser174 occurred despite the depletion of AURKA or the inhibition of its activity. More often, CDK1 activated by speedy/RINGO (independently or in cooperation with AURKA) is proposed to be responsible for the CPEB1 activating phosphorylation in *Xenopus* oocytes [21]-. In the mammalian system, Ca^2+^/calmodulin-dependent protein kinase II (CaMKII) has been found to be responsible for CPEB1 activation in neurons [24]. Thus the relationship between AURKA activity and mRNA cytoplasmic polyadenylation needs to be clarified.

In this study, we show that the cytoplasmic polyadenylation of both short and long forms of porcine *cyclin B1* mRNA precedes CPEB1 degradation. We therefore explored the effects of AURKA inhibition on meiotic resumption of porcine oocytes using MLN8237 and we have found that the MLN8237-treated oocytes remain arrested in the late diakinesis-like stage and that they cannot reach the metaphase I stage. However, neither *cyclin B1* mRNA polyadenylation nor its translation is impaired in these oocytes. Using dual-luciferase assay, we further show that the inhibition of AURKA kinase does not prevent the translation of other CPE-containing mRNAs. Finally, using *in vitro* kinase assay, we demonstrate that CPEB1 is phosphorylated at Thr172 and/or Ser178 during oocyte meiotic maturation despite the inhibition of AURKA.

## Materials and Methods

### Oocyte collection and in vitro maturation (IVM)

Porcine ovaries from non-cycling gilts were collected at a commercial slaughterhouse (Jatky Český Brod a.s., Český Brod, CR) and transported in physiological saline at 37 °C to the laboratory. Cumulus-oocyte complexes were aspirated from follicles and matured in M199 medium (Life technologies, Carlsbad, CA, U.S.A.) supplemented with 10% fetal bovine serum (Sigma-Aldrich, St-Louis, MO, U.S.A.) and 0.8 IU/mL P.G. 600 (Intervet). IVM was performed at 38.5 °C in a humidified atmosphere of 5% CO_2_ for 12 to 44 h. Oocytes were denuded, washed and stored at −80 °C until use. For evaluation of maturation, denuded oocytes were fixed in ethanol:acetic acid solution (3:1 v/v) for 48 h. Staining was performed with 1% orcein in 50% aqueous acetic acid and 1% sodium citrate followed by washing with 40% acetic acid. Oocytes were observed and photographed under a phase-contrast microscope (Carl Zeiss, Jena, Germany).

### Drug treatment

For inhibition of AURKA activity, MLN8237 (Alisertib; Selleck Chemicals, Houston, TX, U.S.A.) was added to the IVM medium at concentrations of 1, 5 and 10 µM. Cumulus-oocyte complexes were cultured for 44 h to evaluate the effects of MLN8237 on meiotic maturation. For the western blot analysis, immunocytochemistry and poly(A)-test, oocytes were cultured in the presence of the inhibitor for 28 h, for the dual-luciferase assay, oocytes were collected after 3, 24 and 28 h of IVM.

### Poly(A)-test

The poly(A)-test was performed as described by Sallés and Strickland [25] with minor modifications. Total RNA was isolated from groups of 50 oocytes using RNeasy Micro Kit (74004; Qiagen). For the reverse transcription, SuperScript III Reverse Transcriptase (Life Technologies) was used. PCR was performed with following primers: Oligo(dT)-Anchor 5′-GCG AGC TCC GCG GCC GCG TTT TTT TTT TTT-3′, cyclin B1 primer 5′-GCA TTT TCT TCG GAG AGC ATC CAA GAT T-3′ (amplification of both short and long *cyclin B1* 3′UTRs) and cyclin B1 long 3′UTR primer 5′-CTC ATT TGA ATG TGG CTA TTT CCC ACT TGA GG-3′ (specific for the long *cyclin B1* 3′UTR). cDNA was subjected to electrophoresis in 5% polyacrylamide gel and stained with GelRed (Biotium, Hayward, CA, U.S.A.). Gels were observed and photographed by Kodak Gel Logic 100/200 Camera (Carestream Health, Inc., Rochester, NY, U.S.A.), K. G. L. integrated illuminator cabinet (Carestream Health, Inc.) and KODAK MI SE software (v. 4.5.0.; Carestream Health, Inc.).

### Western blot analysis

Oocytes were lysed in 10 µL of 1 × Reducing SDS Loading Buffer (Cell Signaling Technology, Danvers, MA, U.S.A.) and heated at 100 °C for 5 min. Proteins were separated by SDS-PAGE and transferred to Immobilon P membrane (Millipore, Bedford, MA, U.S.A.) using a semidry blotting system (Biometra GmbH, Goettingen, Germany) for 25 min at 5 mA/cm^2^. Membranes were blocked, depending on the used antibody, in 10% gelatin, 2.5% or 5% skimmed milk dissolved in 0,05% Tween-Tris-buffer saline (TTBS), pH 7.4 for 1 h. After a brief washing in TTBS, membranes were incubated at 4 °C overnight with the following primary antibodies: CPEB (H-300) (sc-33193; Santa Cruz Biotechnology, Dallas, TX, U.S.A.), cyclin B1 Ab-1 (Clone V152) (MS-338; Thermo Scientific, Fremont, CA, U.S.A), phospho-Aurora A (Thr288)/Aurora B (Thr232)/Aurora C (Thr198) (#2914; Cell Signaling Technology), phospho-TACC3 (Ser558) (#8842; Cell Signaling Technology), Anti-GAPDH (G9545; Sigma-Aldrich), monoclonal anti-β-Tubulin antibody (T4026; Sigma-Aldrich). The membranes were then washed 3 × 10 min in TTBS and incubated with a horseradish peroxidase-conjugated donkey anti-rabbit or anti-mouse IgG antibody (Jackson Immuno Research, Suffolk, UK) for 1 h at room temperature. Proteins were visualized using an ECL-plus detection system (GE Healthcare, Chalfont St Giles, Bucks, UK) according to the manufacturer's instructions. Films were scanned using a GS-800 Calibrated Densitometer (Bio-Rad, Hercules, CA, U.S.A) and the results were quantified using Quantity One 1-D Analysis Software (Bio-Rad).

### Immunocytochemistry

Oocytes were fixed in 4% paraformaldehyde (PFA) in PBS for 30 min, permeabilized for 15 min in 0.1% Triton X-100, and incubated overnight at 4 °C with primary antibody against phospho-Aurora A (Thr288) (NB100-2371; Novus Biologicals, Littleton, CO, U.S.A.). After washing, oocytes were incubated for 1 h with an Alexa Fluor conjugated anti-rabbit antibody (Molecular Probes). Samples were visualized using an inverted confocal microscope in 16 bit depth (TCS SP5; Leica Microsystems GmbH, Wetzlar, Germany). Images were assembled in Photoshop CS3.

### Synthesis and microinjection of cRNA

The pIVT plasmid containing firefly luciferase with mouse *cyclin B1* 3′UTR was a kind gift from Prof. Shin Murai, Department of Biochemistry, Toho University School of Medicine, Tokyo, Japan [26]. The pRL-EMCV plasmid was a kind gift from Martin Bushell Medical Research Council Toxicology Unit, Leicester, UK [27]. Complementary RNAs (cRNAs) were synthesized from linearized plasmids using T7 RNA polymerase and the mMESSAGE mMACHINE kit (Ambion, Austin, TX, U.S.A.) according to the manufacturer's instructions. The pRL-EMCV cRNA was polyadenylated by the Poly(A) Tailing Kit (Ambion) according to the manufacturer's instructions. cRNA was purified using RNeasy Mini kit (Qiagen). The final cRNA concentration was determined by spectrophotometry (NanoDrop, Wilmington, DE, U.S.A.).

Porcine oocytes were microinjected as was described previously [28]. Briefly, isolated oocytes were denuded and kept in supplemented M199 medium without hormones for 1 h at 38.5 °C. Injections were done in drops of manipulation medium using a MIS-5000 micromanipulator (Burleigh, EXFO Life Sciences, U.S.A.) and PM 2000B4 microinjector (MicroData Instrument, U.S.A.). Approximately 5 pL of cRNA was injected into the cytoplasm of each oocyte. The oocytes were transferred into medium with or without 1 µM MLN8237 and cultivated for 3, 24 and 28 h.

### Oocyte viability assay

To exclude dead oocytes being collected for further analyses, we used trypan blue staining. Microinjected oocytes after the IVM in the absence or presence of the inhibitor were stained with 0.1% trypan blue (Beckman-Coulter) for 5 min. Dead oocytes (blue) were discarded and only live oocytes were used for the dual-luciferase assay.

### Dual-luciferase reporter assay

The cRNA of firefly luciferase with *cyclin B1* 3′UTR (0.5 pg/oocyte) and *Renilla* luciferase (0.25 pg/oocyte) were co-injected into GV-stage oocytes as described above. The oocytes were cultured *in vitro* for 3, 24 and 28 hours in the absence or presence of 1 µM MLN8237. Four live oocytes from each group were collected. The oocytes were lysed in 5 µL of Passive Lysis Buffer and stored at −80 °C until luciferase activity was measured by the Dual-Luciferase Assay System (Promega, Madison, WI, U.S.A.) according to manufacturer's instructions. Signal intensities were measured using a Glomax 20/20 Luminometer (Promega). Luciferase activity of firefly luciferase was normalized to that of *Renilla* luciferase.

### In vitro kinase assays

Phosphorylation of CPEB1 and LATS2 was measured in oocyte extracts via their capacity to phosphorylate external substrate peptides derived from CPEB1 and LATS2 sequence: RRSRLDTRPILDSRSS (underlined T corresponding to CPEB1 Thr172 activatory site) and YQKALREIRYSLLPFANESGT (underlined S corresponding to LATS2 Ser83 known to be phosphorylated by AURKA [29]), which were covalently bound on the matrix composed of a “lysine tree” allowing the binding of multiple peptide molecules (synthesized by Apigenex, Prague, CR). The *in vitro* kinase assay was a modified version of the method published earlier [30], [31], briefly: at each time interval during the culture, 20 oocytes per sample were lysed in 5 µL of homogenization buffer containing 40 mM MOPS, pH 7.2, inhibitors of phosphatases and proteases, by three rounds of freezing/thawing on dry ice. When the effect of MLN8237 was examined, the inhibitor was added in the concentration of 2 to 10 µM directly to the reaction buffer. The kinase reaction was initiated by addition of 5 µL of kinase buffer containing 20 mg/mL of one of the above-mentioned peptide together with 10 mCi/mL [γ-^32^P] ATP (Amersham Pharmacia Biotech). The reaction was stopped after 30 min by the addition of SDS-PAGE sample buffer and boiling for 3 min. After SDS-PAGE on 15% gel, the gels were stained with Coomassie Blue R250, destained overnight and dried. The phosphorylated substrates were visualized by autoradiography and analyzed using a BAS 2500 PhosphoImager (Fujifilm, Japan).

### Statistical analysis

Pearson's product-moment correlation coefficient was counted for the correlation between the measured cyclin B1 expression in oocytes cultivated for 28 h and the concentration of MLN8237 in the medium. The data from this experiment were analyzed also by ANOVA with the Dunnett's test in GraphPad Prism 6.03. Data from the dual-luciferase assay were analyzed using t-test with Welch's correction.

## Results

### Porcine cyclin B1 mRNA polyadenylation is independent of CPEB1 degradation

**Figure 1:**
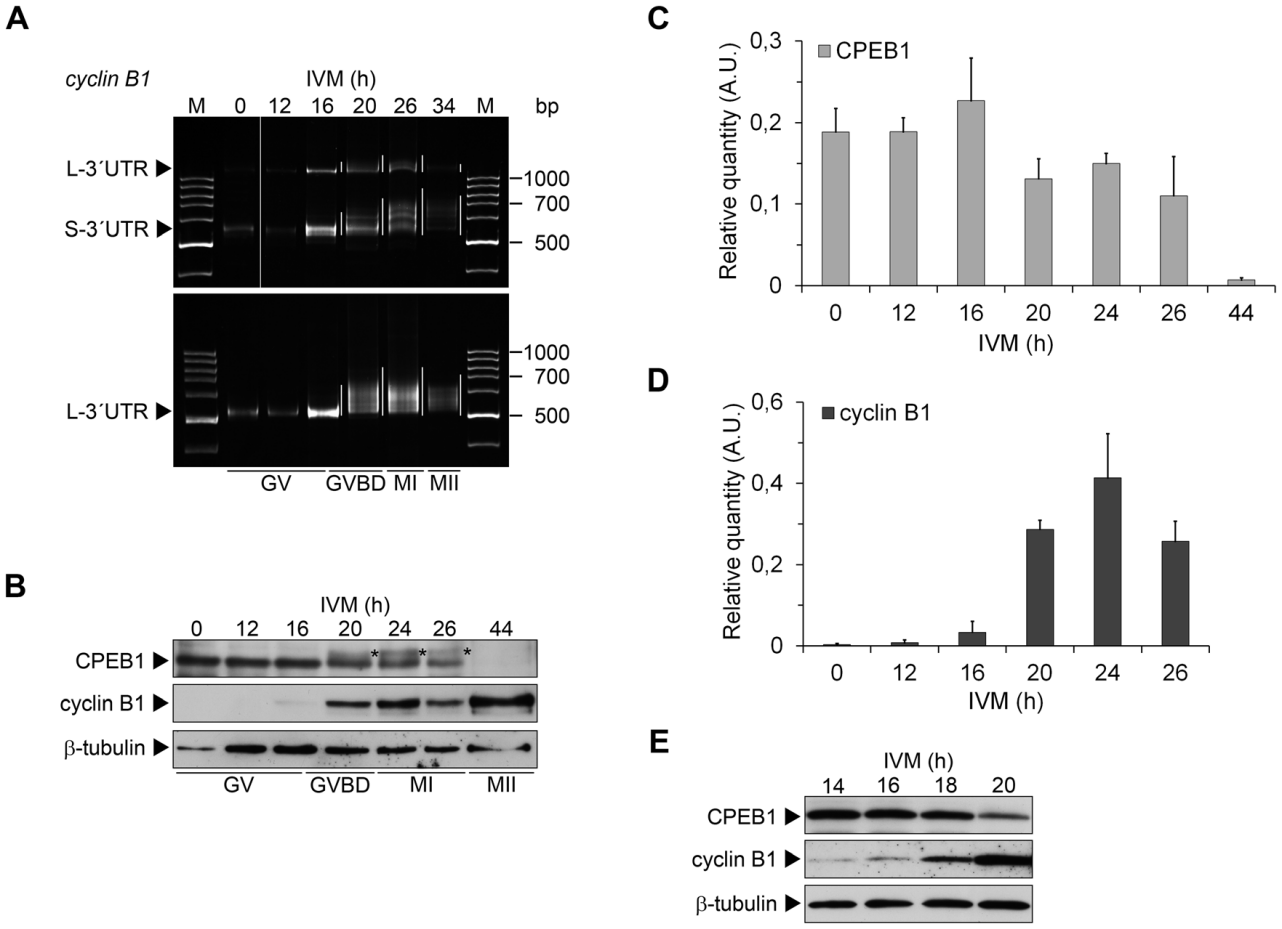
Polyadenylation of *cyclin B1* mRNA and a comparison of cyclin B1 and CPEB1 expression during IVM of porcine oocytes. (A) Polyadenylation of *cyclin B1* long (L-3′UTR) and short (S-3′UTR) mRNAs was examined by poly(A)-test in oocytes collected in different times of *in vitro* maturation (IVM). Corresponding maturation phases are figured below. The polyadenylation is highlighted by white lines next to each lane. (B) Oocytes collected after 0 to 44 h of IVM were subjected to western blot analysis of CPEB1 and cyclin B1 using specific antibodies. Detection of β-tubulin was used as a loading control. The phosphorylated form of CPEB1 is marked (*). (C) The protein expression of CPEB1 from three independent experiments was quantified using Quantity one software. The density of individual band was normalized to the total density of examined bands and the values of CPEB1 and phosphorylated CPEB1 were summed. The values represent the means ± SEM. (D) The protein expression of cyclin B1 from two independent experiments was quantified using Quantity one software. The density of individual band was normalized to the total density of examined bands. The values represent the means ± SEM. (E) Oocytes collected after 14, 16, 18 and 20 h of IVM were subjected to western blot analysis of CPEB1 and cyclin B1 using specific antibodies. Detection of GAPDH was used as a loading control.

In *Xenopus* oocytes, the *cyclin B1* mRNA polyadenylation occurs at metaphase I and depends on the partial degradation of CPEB1 [7]. We studied the polyadenylation of *cyclin B1* mRNA during meiotic maturation of porcine oocytes, from GV to metaphase II, using poly(A)-test (PAT). We observed that both short and long form of *cyclin B1* mRNA were gradually polyadenylated (Figure 1A). These forms differ in a number of regulatory elements; therefore the timing of their translation may be different [32]. However, in our conditions, the polyadenylation of both short and long form of *cyclin B1* mRNA, as well as the expression of cyclin B1, was evident after 16 hours of *in vitro* maturation (IVM) (Figure 1). At this time, most of the oocytes (92%) were still at the GV stage.

We observed the CPEB1 phosphorylation by a shift in its electrophoretic mobility starting at 20 h of IVM (Figure 1B). Analogous phosphorylation in *Xenopus* oocytes is required for CPEB1 degradation and it depends on both CDK1 and PLK1 activities [7], [9]. In porcine oocytes, the inhibition of CDK1 activity by roscovitine during the course of meiotic maturation prevented CPEB1 phosphorylation and degradation (data not shown). Although porcine CPEB1 was slightly degraded after GVBD (20 h of IVM), the majority of the protein was stable until metaphase I (starting from 24 h of IVM), after which it was massively destroyed (Figure 1C).

To follow the cyclin B1 expression more precisely during the critical time interval, we collected the oocytes before and around GVBD in the interval of 2 hours. Low expression of cyclin B1 was evident already after 14 h of IVM and the signal gradually increased in following time intervals (Figure 1B,C,D). On the contrary, CPEB1 was stable until 16 to 18 hours (Figure 1B,C,E). This result documents that the polyadenylation of *cyclin B1* mRNA and its translation are likely to precede CPEB1 phosphorylation by CDK1 and subsequent CPEB1 destruction. Therefore, our data suggest that the mechanism of *cyclin B1* mRNA polyadenylation regulation in porcine oocytes probably differs from that in *Xenopus* oocytes and is dependent on CPEB1 activating phosphorylation rather than on its degradation.

### Verification of MLN8237 effect on AURKA kinase activity in porcine oocyte model system

MLN8237 is a highly selective AURKA inhibitor [33]. Although it has been previously shown to inhibit AURKA activity in a number of different types of somatic cells [34]–[37], it has not been used in mammalian oocytes yet. To verify its potential to inhibit AURKA kinase activity in our model system, we performed following experiments: Firstly, utilizing a western blot analysis, we monitored the effect of MLN8237 on phosphorylation of the transforming, acidic coiled-coil containing protein 3 (TACC3), a known AURKA substrate, which was used previously to detect AURKA activity both in somatic cells [19], [38] and in mouse oocytes [39]. Our results show that MLN8237 at a concentration as low as 1 µM was able to block AURKA activity and substantially reduced TACC3 phosphorylation (Figure S1A). The remaining phosphorylation of TACC3 visible in oocyte samples treated with MLN8237 could be most likely attributed to phosphorylation by other protein kinases, such as CDK1 or ATM (predicted to phosphorylate TACC3 by Scansite 2.0 database [40]). Secondly, we performed an *in vitro* kinase assay using a peptide derived from another known substrate of AURKA, the large tumor suppressor kinase 2 (LATS2) [29], with a sequence encompassing Ser83 as an external substrate (as described in the Materials and methods section). The results (Figure S1B) showed the phosphorylation of the peptide at different stages of oocyte maturation, as well as the inhibition of this phosphorylation in the samples where 2 to 10 µM MLN8237 was added to the extracts. Moreover, MLN8237 also abolished the phosphorylation of the peptide when active recombinant AURKA was used as a positive control. Peptide phosphorylation, visible in the extracts from oocytes treated with MLN8237 for 20 h *in vivo,* could be attributed to the mode of action of the inhibitor. MLN8237 inhibited AURKA activity *in situ*, but it did not prevent its activation and as such it could not prevent the peptide phosphorylation in kinase reaction with no inhibitor added. Altogether, the above-mentioned results document clearly that MLN8237 was able to inhibit AURKA activity and to prevent phosphorylation of its downstream targets in maturing porcine oocytes.

### Inhibition of AURKA activity by MLN8237 results in a late diakinesis-like arrest

**Figure 2:**
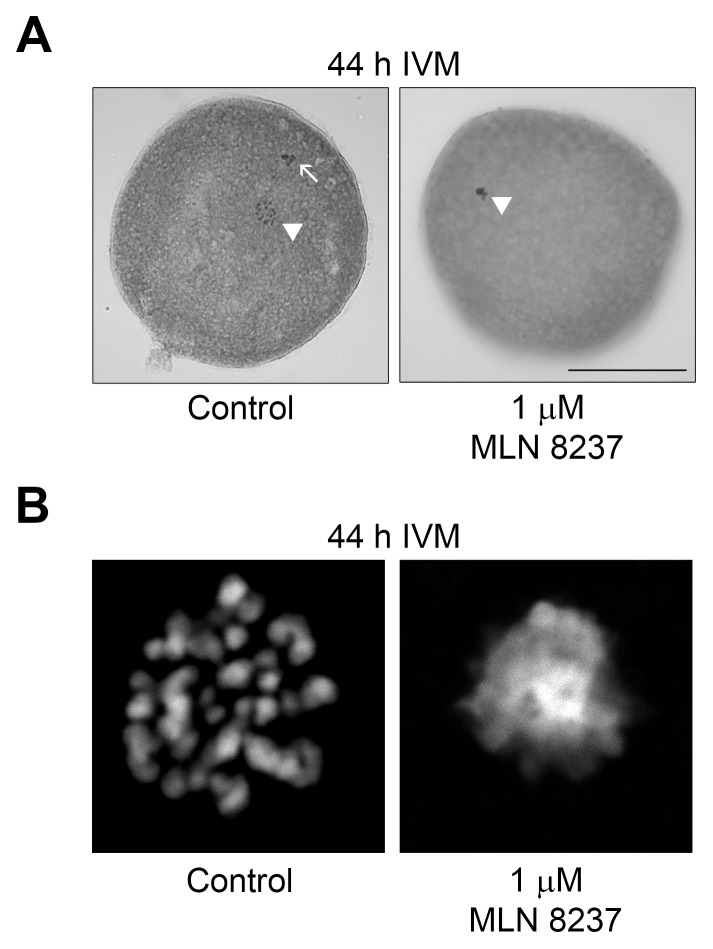
Morphological aspect of oocytes treated with MLN8237. Representative examples of morphological appearance of oocytes cultivated for 44 µM MLN8237. (A) Oocytes were fixed with ethanol:acetic acid (3:1) and stained by orcein. Sets of chromosomes are marked with arrowheads, a polar body is marked with and arrow. (B) Detail of chromosome configuration visualized by DAPI.

To further study the regulatory mechanism of *cyclin B1* mRNA polyadenylation and the role of AURKA in this process, we inhibited AURKA kinase activity in oocytes by adding MLN8237 at different concentrations ranging from 1 to 10 µM to the cultivation medium. At low concentrations of the inhibitor, the oocytes underwent GVBD but they were unable to reach metaphase I, and they became arrested in late diakinesis-like configuration of chromosomes even after 44 h of IVM (Figure 2). With increasing concentrations of MLN8237 in the medium, a portion of oocytes was arrested at earlier phases of maturation (GV IV to GV I) (Figure S2). The 1 µM MLN8237 was sufficient to block AURKA activity (Figure S1) and did not inhibit GVBD, therefore we used this concentration in the majority of our experiments.

**Figure 3:**
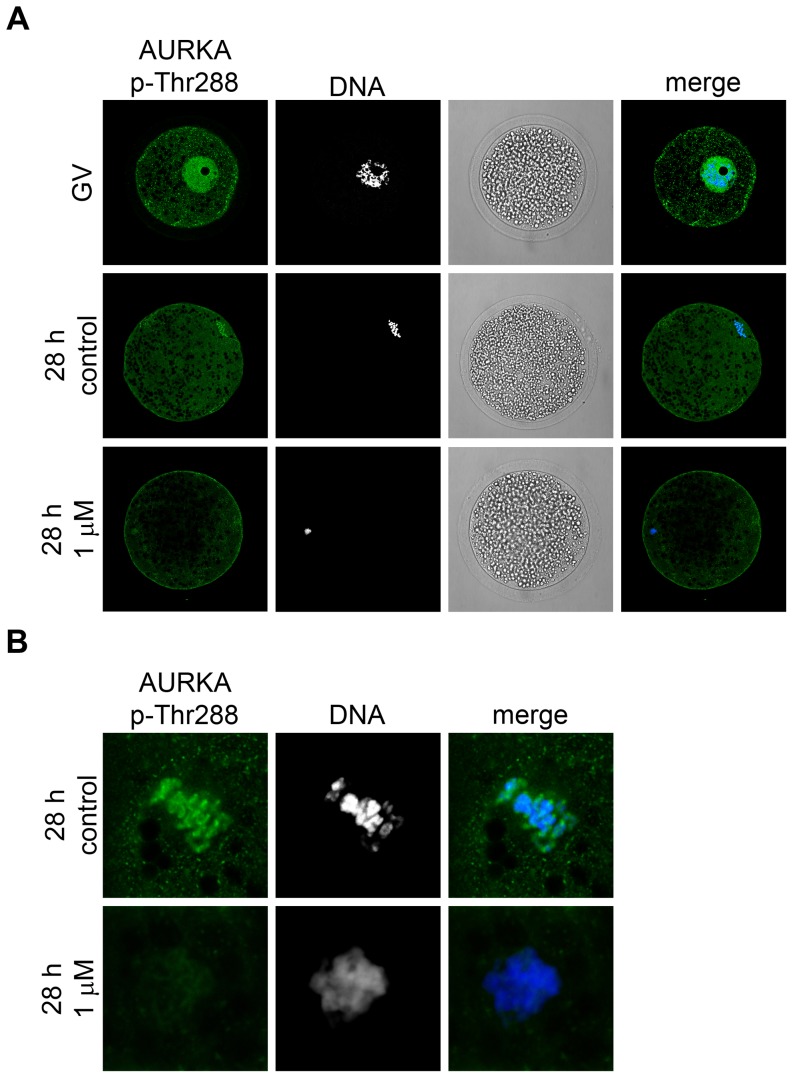
Detection of AURKA (Thr288) phosphorylation. (A) Fluorescent images of AURKA p-Thr288 localization detected by specific antibody in the GV- and MI-stage oocytes (28 h of IVM) and the oocytes cultivated for 28 h in the presence of 1 µM MLN8237. DNA is stained by DAPI. (B) A detail of chromosomes stained by AURKA p-Thr288 antibody in control MI-stage oocytes (28 h of IVM) and oocytes cultured for 28 h in medium supplemented with 1 µM MLN8237. Representative images from two independent experiments.

**Figure 4:**
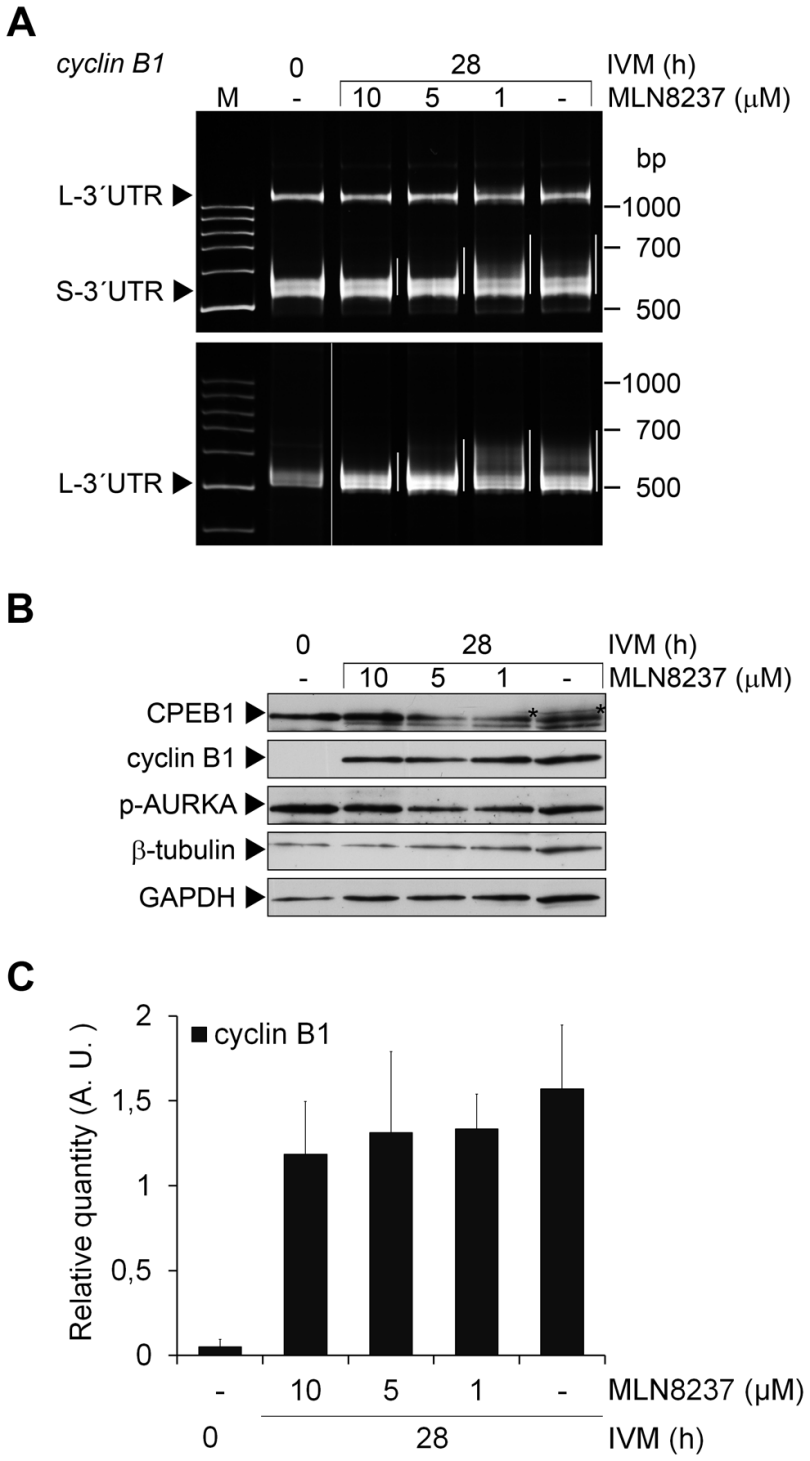
*Cyclin B1* mRNA polyadenylation and cyclin B1 expression after AURKA inhibition. (A) Polyadenylation of short (S-3′UTR) and long (L-3′UTR) forms of the *cyclin B1* mRNA was examined by poly(A)-test in oocytes collected before and after 28 h of IVM in media supplemented with stated concentrations of MLN8237. The polyadenylation is highlighted by white lines next to each lane. (B) Oocytes collected before IVM and after 28 h of IVM in media supplemented with stated concentrations of MLN8237 were subjected to western blot analysis of CPEB1, cyclin B1 and phospho-AURKA using specific antibodies. β-tubulin and glyceraldehyde-3-phosphate dehydrogenase (GAPDH) were used as loading controls. The phosphorylated form of CPEB1 is marked (*). (C) The protein expression of cyclin B1 from six independent experiments was quantified using Quantity one software. The density of individual band was normalized to the total density of examined bands and to β-tubulin. The values represent the means ± SEM. No significant difference was detected between the control oocytes after 28 hours of IVM and the oocytes treated with different concentrations of MLN8237, P>0.05.

We then monitored the localization and activity of AURKA via its phosphorylation at Thr288. To observe the localization of active AURKA, we performed the immunocytochemistry with a specific anti-phospho-Thr288 AURKA antibody. In the GV-stage oocytes, phosphorylated AURKA was present predominantly in the nucleus. After 28 h of IVM, when the oocytes were in the metaphase I stage, phosphorylated AURKA was associated with the chromosomes (Figure 3A). In MLN8237-treated oocytes, AURKA phosphorylation at Thr288 was only slightly reduced (Figure 3B), which suggested that the inhibitor was not able to reduce the phosphorylation present already in the GV-stage oocytes. We also used another phosphorylation-specific antibody to detect the degree of AURKA phosphorylation by western blot. The signal was present even in the GV-stage oocytes and did not substantially change in the oocytes cultivated in the presence of MLN8237 (Figure 4). However, it should be mentioned that the inhibitory mechanism of MLN8237 is not dependent on the AURKA phosphorylation, i.e. it inhibits the activity but not the activation of AURKA.

### Cytoplasmic polyadenylation and translation of cyclin B1 mRNA is not affected by AURKA inhibition

To investigate the effect of AURKA inhibition on polyadenylation of *cyclin B1* mRNA, we examined the polyadenylation status of *cyclin B1* mRNA in oocytes cultivated with 1 to 10 µM MLN8237 in the medium by PAT. Although the polyadenylation was less evident in oocytes cultivated with higher concentrations of the inhibitor (10 µM, 5 µM), when 1 µM MLN8237 was used, the polyadenylation was similar to the polyadenylation observed in the control group (Figure 4A). We assume that the effect of higher concentration is caused by the non-specific inhibition of maturation rather than the inhibition of AURKA. Moreover, cyclin B1 protein expression was not prevented even by the 10 µM MLN8237 (Figure 4B). There was no correlation between the cyclin B1 protein expression and the concentration of MLN8237 according to the correlation coefficient, which was close to zero value (R  =  -0.145), and the differences between the expression of cyclin B1 in the control group and in the groups treated with MLN8237 were not significant, P>0.05 (Figure 4C).

**Figure 5:**
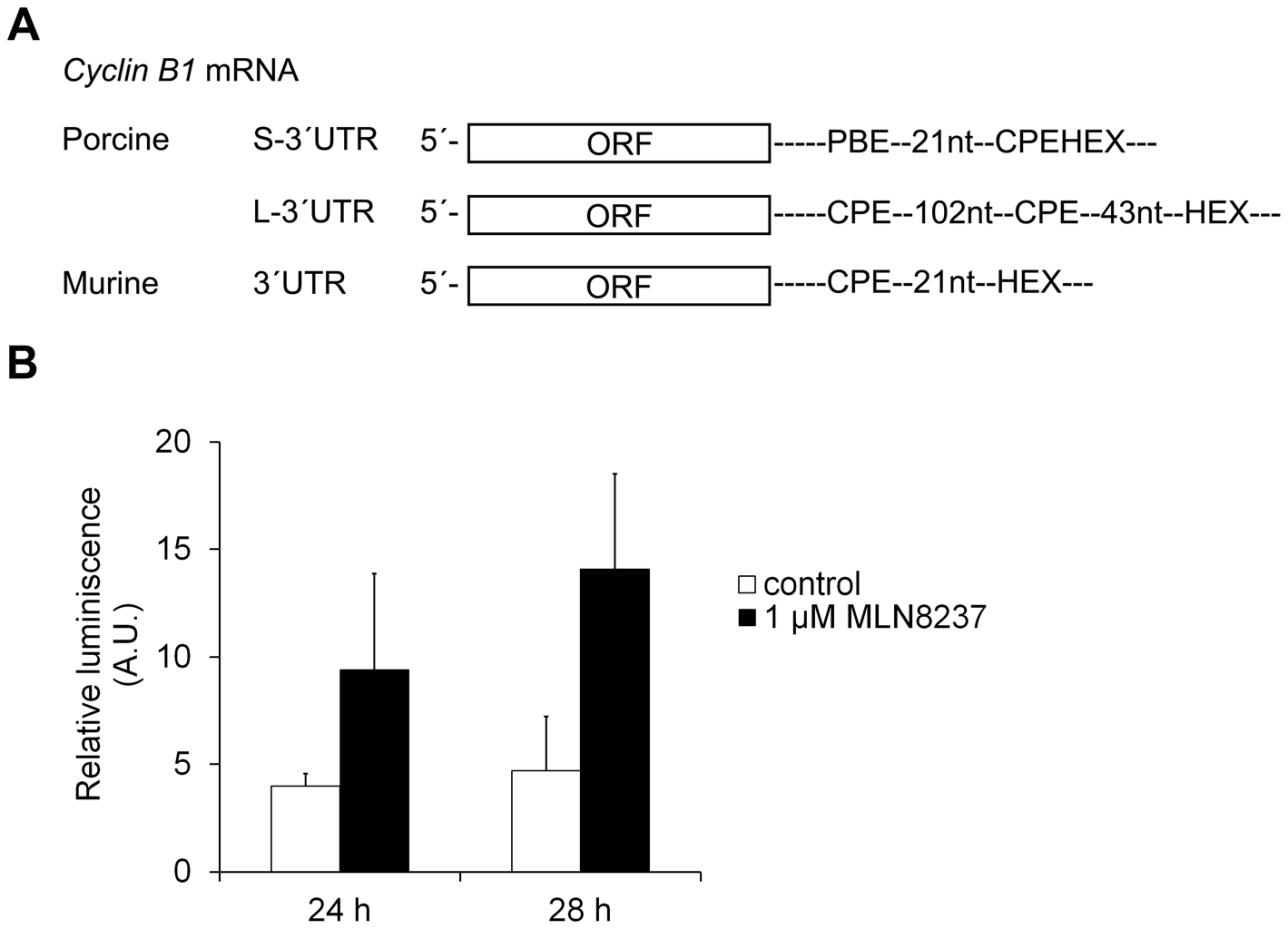
Translation of luciferase reporter with *cyclin B1* 3′UTR in the presence or absence of MLN8237. (A) Schematic representation of porcine and murine *cyclin B1* 3′UTRs. ORF – open reading frame; CPE – cytoplasmic polyadenylation element; HEX – hexanucleotide, polyadenylation signal; PBE – Pumilio binding element. (B) A non-polyadenylated cRNA containing firefly luciferase open reading frame with murine *cyclin B1* 3′UTR was co-injected with polyadenylated *Renilla* luciferase cRNA as an internal injection control into GV-stage oocytes. Oocytes were cultivated for 24 and 28 hours in an inhibitor free medium (control) or the medium supplemented with 1 µM MLN8237. The firefly luciferase activity was normalized to the *Renilla* luciferase activity. The values are represented as means ± SEM. Data were subjected to t-test with Welch's correction. The differences between control and treated groups are not significant, P>0.05.

To expand our findings to other CPE-containing mRNAs, we microinjected a non-polyadenylated firefly luciferase reporter containing murine *cyclin B1* 3′UTR [26] (Figure 5A) together with a control *Renilla* luciferase reporter [27] into the oocytes. The activity was measured after 3, 24 and 28 hours of IVM with or without MLN8237. No luciferase activity was measured after 3 hours of IVM (data not shown). The firefly luciferase activity normalized to *Renilla* luciferase activity increased during the maturation both in the control and MLN8237-treated oocytes (Figure 5B). This result supports the hypothesis that the activity of AURKA is not necessary for the cytoplasmic polyadenylation of CPE-containing mRNAs in porcine oocytes.

### CPEB1 is gradually phosphorylated at Thr172 and/or Ser178 during oocyte maturation independently of AURKA activity

**Figure 6:**
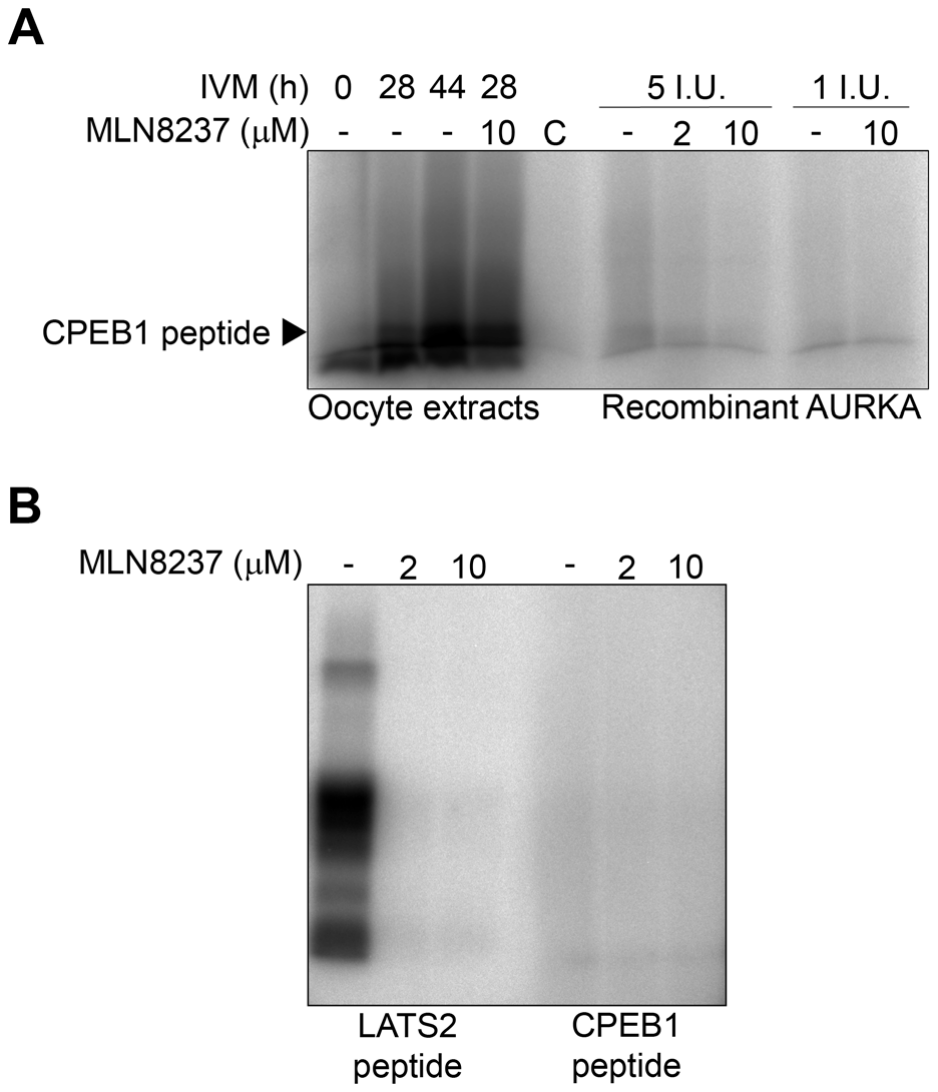
*In vitro* kinase assay – CPEB1 phosphorylation. (A) Oocytes were cultivated in control medium for 0, 28 or 44 h, Thereafter, *in vitro* kinase assay with CPEB1 peptide as an external substrate was performed in extracts prepared from 20 oocytes. MLN8237 was added to MI (28 h of IVM) oocyte extracts in final concentration of 10 µM in reaction buffer. Alternatively, recombinant AURKA (1 and 5 I.U. per assay) was used instead of oocyte extracts with or without MLN8237 in the concentration of 2 and 10 µM. The extracts were separated by SDS-PAGE and the phosphorylated substrates were visualized by autoradiography and analyzed using a BAS 2500 PhosphoImager. (B) *In vitro* kinase assay utilizing recombinant AURKA (1 I.U.) with or without MLN8237 in the concentration of 2 and 10 µM showing its activity towards either CPEB1 peptide as an external substrate, or LATS2 peptide used as a positive control. Phosphorylated substrates were separated by SDS-PAGE, visualized by autoradiography and analyzed using a BAS 2500 PhosphoImager.

To determine the possible involvement of AURKA in CPEB1 phosphorylation we designed an *in vitro* kinase assay using a peptide derived from the porcine CPEB1 sequence encompassing Thr172 as an external substrate, as described in the Materials and methods section. The peptide was not phosphorylated in lysates produced from the GV-stage oocytes but was gradually phosphorylated in lysates produced from oocytes collected after 28 or more hours of IVM (Figure 6A) indicating that the kinase responsible for this phosphorylation was not active in the GV-stage oocytes. Phosphorylation profile of CPEB1-derived peptide is therefore different from the one observed using the LATS2 peptide. Moreover, unlike the LATS2 peptide, phosphorylation of CPEB1 was not affected when MLN8237 was added directly to the extracts in the *in vitro* kinase assay. Finally, we used active recombinant AURKA in the *in vitro* assay in order to detect its role in phosphorylating the CPEB1 peptide. As we show in the Figure 6A,B, the recombinant AURKA was not able to phosphorylate CPEB1 peptide. On the other hand, under the same kinase assay conditions, it was able to phosphorylate the peptide derived from the known AURKA substrate LATS2 and this phosphorylation could be effectively inhibited by MLN8237 (Fig. 6B, S1B). Altogether, these results suggest that other protein kinase than AURKA is responsible for CPEB1 Thr172 phosphorylation in porcine oocytes. The nature and/or the identity of this kinase are yet to be revealed.

## Discussion

In this study, we examined the relationship between CPEB1 activation and degradation, AURKA activity and the *cyclin B1* mRNA cytoplasmic polyadenylation and expression.

It has been shown in many species and different cell types as well that cyclin B1 synthesis is regulated by the cytoplasmic polyadenylation of its mRNA [32], [41]–[44]. We have detected the polyadenylation of both short and long form of *cyclin B1* mRNA during meiotic maturation of porcine oocytes starting at 16 h of *in vitro* maturation (IVM). This result is in agreement with previous findings, although Zhang et al. [32] showed that the polyadenylation of *cyclin B1* mRNA in porcine oocytes starts at 24 h, This difference in the timing of polyadenylation may be caused by the use of a more sensitive electrophoretic system in our case (polyacrylamide gel vs. agarose gel) together with the different speed of IVM in different conditions (MI – 30 h vs. 26–28 h in our conditions).

Regarding the regulation of cytoplasmic polyadenylation by *cis*-elements within the 3′untranslated region (3′UTR) of mRNAs, mRNA with short 3′UTR contains one CPE overlapping with polyadenylation signal, the hexanucleotide AAUAAA and one Pumilio binding element (PBE) in the distance of 32 nts from the hexanucleotide. According to Piqué et al. [12], the overlapping CPE is not functional in translational activation and the binding of CPEB1 is very weak. The PBE is necessary for the single overlapping CPE to bind CPEB1. On the contrary, the long 3′UTR contains six CPEs. The closest distance between CPE and the hexanucleotide is 43 nts, the second CPE is 102 nts distant from the first one (Figure 5). Since the CPE in the proximity of 3 to 45 nts from the hexanucleotide acts as a strong translational activator and the second CPE is too distant for the translational repression that is under the control of CPEB1 dimerization [12], the long form of *cyclin B1* mRNA should be theoretically polyadenylated soon after the beginning of oocyte maturation. Therefore, this polyadenylation should be dependent on CPEB1 activating phosphorylation. This is in a good agreement with our results showing that the long form of *cyclin B1* mRNA is polyadenylated before GVBD and that there is no difference in the timing of polyadenylation between the long and short form.

Nishimura et al. [45] have shown that the overexpression of CPEB1 in porcine oocytes increases cyclin B1 expression and the rate of meiotic resumption. Moreover, the mutated form of CPEB1, which cannot be phosphorylated at both Thr172 and Ser178 and acts as a dominant negative mutant, inhibits the cyclin B1 synthesis, c-mos activity and meiotic resumption. Our results support these findings by showing that cytoplasmic polyadenylation and translation of *cyclin B1* mRNA precedes CPEB1 phosphorylation and degradation. This suggests that the polyadenylation of porcine *cyclin B1* mRNA is rather independent of CPEB1 degradation, although we cannot rule out the possibility that some partial degradation of CPEB1, which is below our detection limit, already occurs at this time.

In *Xenopus* and mouse oocytes, AURKA has been previously proposed to be the kinase responsible for the CPEB1 activating phosphorylation [13], [14]. In this study, we have used a highly selective inhibitor of AURKA, MLN8237, to examine the effect of AURKA inhibition on meiotic maturation and mRNA polyadenylation. Both in mitotic and meiotic cells, the inhibition or depletion of AURKA causes defects in spindle assembly, failure of chromosome alignment and metaphase arrest [15]–[17], [19]. The arrest is transient as the cells subsequently undergo aberrant cell division leading to death or senescence [46]. Only little is known regarding the effects of small molecule inhibitors of AURKA on meiotic progression. As far as we know, this is the first time the effects of MLN8237 on meiosis were examined, although its effects are well described in cancer cells [19], [33], [47]. The MLN8237-treated oocytes are arrested in the late diakinesis-like stage suggesting that these oocytes are unable to form the metaphase I plate correctly. This may be related to the reduced level of TACC3 phosphorylation (Figure S1). We previously observed a similar phenotype when oocytes were cultivated with ZM447439, a selective inhibitor of Aurora kinases [48].

Phosphorylation of AURKA at Thr288 is often used as a marker of its activity [49]. We have detected phosphorylated form of AURKA during the period from GV to MI stage. Using immunocytochemistry, we have further observed phosphorylated AURKA in the nucleus of the GV-stage porcine oocytes and at the chromosomes in metaphase I. The localization of AURKA phosphorylated at Thr288 published so far is rather controversial. In prometaphase and metaphase mitotic cells, phosphorylated AURKA has been reported to be localized at centrosomes and spindle poles [50], [51]. In prophase cells, which are similar to GV-stage oocytes arrested in the prophase I of meiosis, the nuclear localization analogous to our observation has been shown [15], [52]. In mouse oocytes, phosphorylated AURKA has not been detected in the GV stage [17], [18], but the total AURKA has been observed in the nucleus of GV-stage oocytes and around the condensed chromatin after GVBD [53]. Finally, in bovine oocytes, phospho-AURKA has been detected in the cytoplasm [54]. Although the oocytes matured in the presence of MLN8237 exhibit a clear phenotype, we have not been able to detect a significant decrease of Thr288 phosphorylation by either western blot or immunocytochemistry (Figure 4). We assume that the inhibitor may not be able to markedly decrease the phosphorylation of AURKA, which is present from the beginning of maturation.

In oocytes treated with MLN8237, polyadenylation of *cyclin B1* mRNA is not affected. The slight inhibitory effect of higher concentrations on the polyadenylation of *cyclin B1* mRNA is likely to be due to the delayed or blocked meiotic progression. Nevertheless, the expression of cyclin B1 protein is not inhibited even in the presence of 10 µM MLN8237. These results are confirmed by the dual-luciferase assay with the luciferase construct containing murine *cyclin B1* 3′UTR with a strong activating CPE in a distance of 21 nts from the hexanucleotide AAUAAA. In mouse oocytes microinjected with this construct, an intensive increase of firefly luciferase activity was observed during maturation [26]. We have observed similar behavior in porcine oocytes when no firefly activity was detected after 3 hours of IVM in contrast to oocytes cultivated for 24 or 28 hours. In accordance with our findings, the relative firefly luciferase activity was not reduced in oocytes cultured in the presence of MLN8237.

Since AURKA phosphorylation at Thr288 and therefore its activity is present already in GV oocytes, we have explored the possibility that AURKA phosphorylates CPEB1 early in the GV stage and in such a case CPEB1 activation and subsequently cytoplasmic polyadenylation would not be inhibited by the inhibitor. However, our results show that this is not the case, because the peptide derived from CPEB1 is not phosphorylated by the GV-stage oocyte lysates even though it is substantially phosphorylated by lysates of oocytes cultivated for 28 h. CPEB1 activation in the early GV stage would also not explain why *cyclin B1* mRNA is polyadenylated shortly before GVBD and not earlier. Moreover as we also show, CPEB1 peptide is not phosphorylated by recombinant AURKA at all suggesting that another kinase than AURKA may be involved in the CPEB1 activatory phosphorylation.

Altogether, our results show that although AURKA plays a crucial role in meiotic progression of oocytes to reach metaphase I, the CPEB1-dependent cytoplasmic polyadenylation is unlikely to be dependent on AURKA activity during meiotic maturation of porcine oocytes. Our further studies with different kinase inhibitors (unpublished) suggest that CDK kinases or MAP kinases could be involved in the regulation of cytoplasmic polyadenylation and CPEB1 activation, which was also proposed by other groups [20]–[23]. However, further analyses have to be performed to confirm these data.

## Supporting Information

Figure S1 **Verification of MLN8237 functionality in porcine oocyte model system.** (A) Oocytes were cultivated in the presence or absence of MLN8237 (1, 5 or 10 µM) and collected after 28 h. The activity of AURKA in the samples was determined using western blot with phospho-TACC3 (Ser558) antibody. (B) *In vitro* kinase assay – LATS2 phosphorylation. Oocytes were cultivated in control medium for 0, 20 or 28 h, or in the presence of 2 µM MLN8237. Thereafter, *in vitro* kinase assay with LATS2 peptide as external substrate was performed in extracts prepared from 20 oocytes. MLN8237 was added to 20 h cultured oocyte extracts in final concentration of 2 or 10 µM in reaction buffer. Alternatively, recombinant AURKA (1 I.U. per assay) was used instead of oocyte extracts with or without MLN8237 in the concentration of 5 and 2 µM. The extracts were separated by SDS-PAGE and the phosphorylated substrates were visualized by autoradiography and analyzed using BAS 2500 PhosphoImager.
(TIF)

Figure S2 **GVBD rate in oocytes treated with MLN8237.** Oocytes were cultivated in the presence or absence of MLN8237 (1, 5 or 10 µM) and collected after 20, 26 and 28 h.
(TIF)
