# Supplementary figures and images for: Aurora Kinase A Is Not Involved in CPEB1 Phosphorylation and cyclin B1 mRNA Polyadenylation during Meiotic Maturation of Porcine Oocytes
Source: PLoS One. 2014 Jul 1;9(7):e101222. doi: 10.1371/journal.pone.0101222 (PMC4077738; doi:10.1371/journal.pone.0101222)

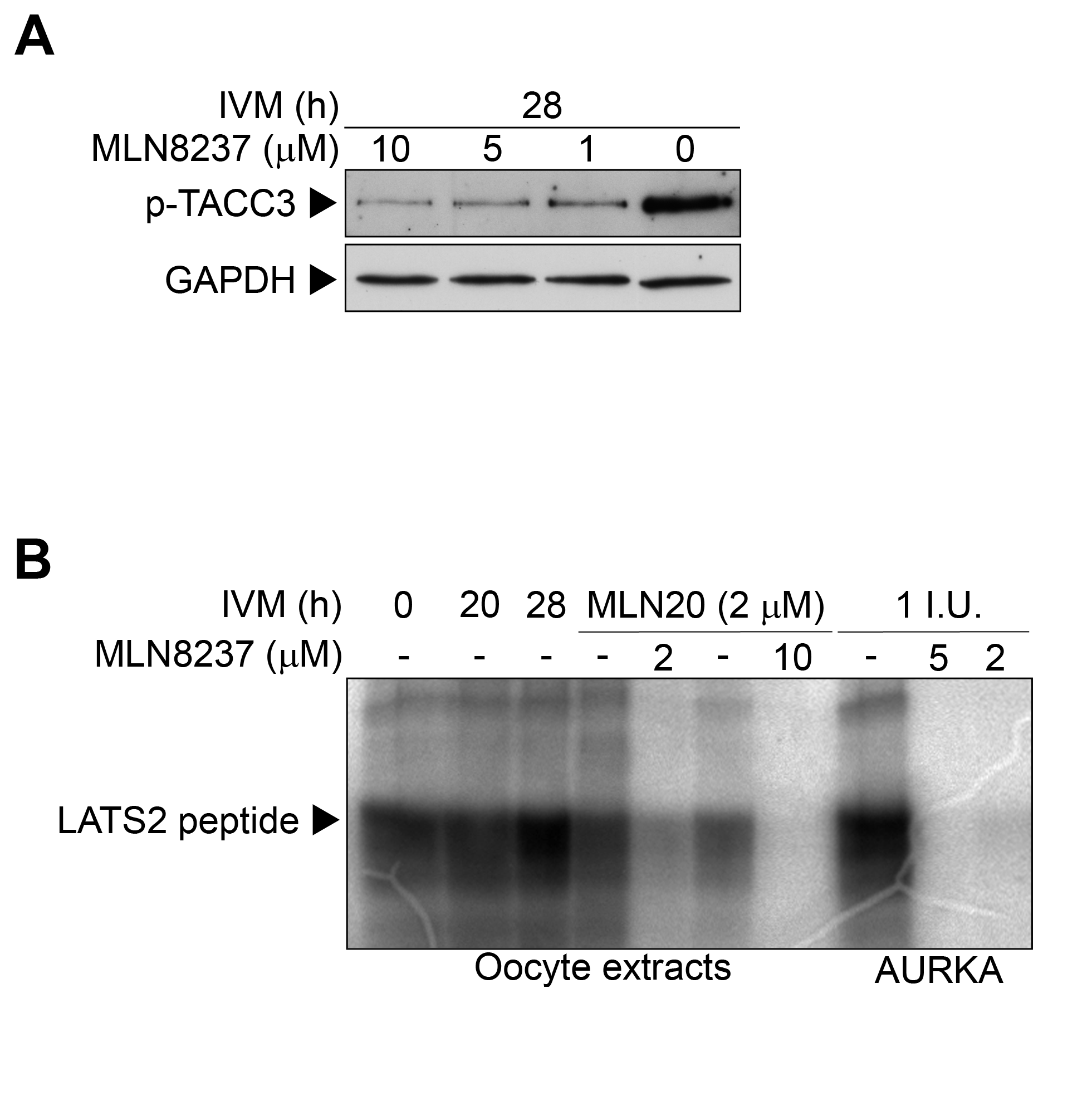

Supplement: Figure S1 — Verification of MLN8237 functionality in porcine oocyte model system. (A) Oocytes were cultivated in the presence or absence of MLN8237 (1, 5 or 10 µM) and collected after 28 h. The activity of AURKA in the samples was determined using western blot with phospho-TACC3 (Ser558) antibody. (B) In vitro kinase assay – LATS2 phosphorylation. Oocytes were cultivated in control medium for 0, 20 or 28 h, or in the presence of 2 µM MLN8237. Thereafter, in vitro kinase assay with LATS2 peptide as external substrate was performed in extracts prepared from 20 oocytes. MLN8237 was added to 20 h cultured oocyte extracts in final concentration of 2 or 10 µM in reaction buffer. Alternatively, recombinant AURKA (1 I.U. per assay) was used instead of oocyte extracts with or without MLN8237 in the concentration of 5 and 2 µM. The extracts were separated by SDS-PAGE and the phosphorylated substrates were visualized by autoradiography and analyzed using BAS 2500 PhosphoImager. (TIF) [file pone.0101222.s001.tif]

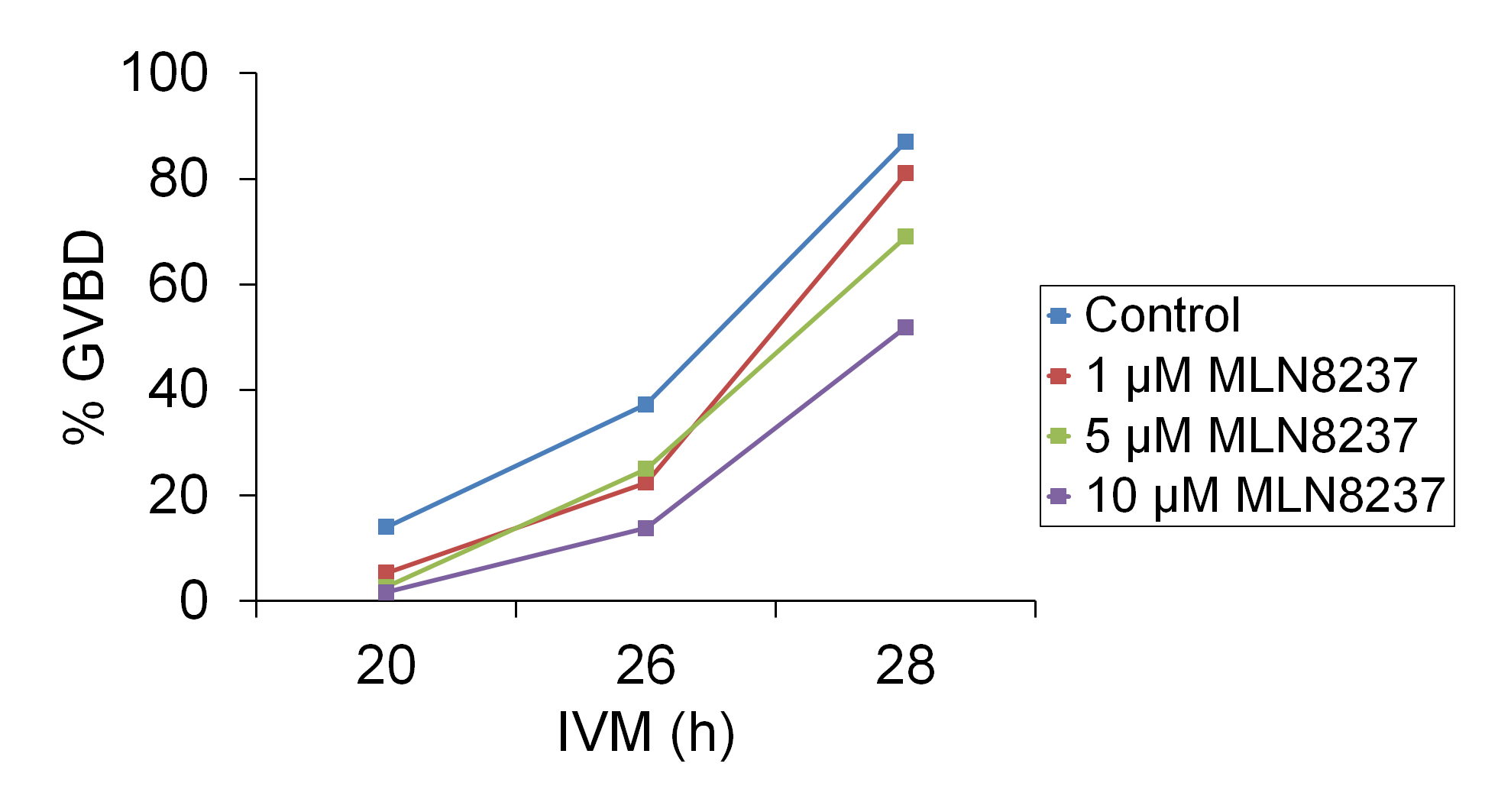

Supplement: Figure S2 — GVBD rate in oocytes treated with MLN8237. Oocytes were cultivated in the presence or absence of MLN8237 (1, 5 or 10 µM) and collected after 20, 26 and 28 h. (TIF) [file pone.0101222.s002.tif]
